# Simplified Classification of Capillary Pattern in Barrett Esophagus Using Magnifying Endoscopy With Narrow Band Imaging: Implications for Malignant Potential and Interobserver Agreement

**DOI:** 10.1097/MD.0000000000000405

**Published:** 2015-01-26

**Authors:** Goichi Uno, Norihisa Ishimura, Yasumasa Tada, Yuji Tamagawa, Takafumi Yuki, Takashi Matsushita, Shunji Ishihara, Yuji Amano, Riruke Maruyama, Yoshikazu Kinoshita

**Affiliations:** From the Department of Gastroenterology and Hepatology (GU, NI, Y. Tada, Y. Tamagawa, SI, YK), Shimane University School of Medicine; Division of Endoscopy (TY), Shimane University Hospital; Department of Pathology (TM, RM), Shimane University School of Medicine, Izumo; Division of Endoscopy (YA), Kaken Hospital, International University of Health and Welfare, Ichikawa, Japan; and Department of Internal Medicine (Y. Tamagawa), University of Texas Southwestern Medical Center, Dallas, TX.

## Abstract

The classification of Barrett esophagus (BE) using magnifying endoscopy with narrow band imaging (ME-NBI) is not widely used in clinical settings because of its complexity. To establish a new simplified available classification using ME-NBI.

We conducted a cross-sectional study in a single-referral center. One hundred eight consecutive patients with BE using ME-NBI and crystal violet (CV) chromoendoscopy, and histological findings were enrolled. BE areas observed by ME-NBI were classified as type I or II on the basis of capillary pattern (CP), and as closed or open type on the basis of a mucosal pit pattern using CV chromoendoscopy; then, biopsy samples were obtained. We evaluated the relation between CP and pit pattern, expression of the factors with malignant potential, percentage of microvascular density, and interobserver agreement.

One hundred thirty lesions from 91 patients were analyzed. Type II CP had more open type pit pattern areas and significantly greater microvascular density than type I. The presence of dysplasia, specialized intestinal metaplasia, expressions of COX-2, CDX2, and CD34, and PCNA index were significantly higher in type II, whereas the multivariate analysis showed that type II was the best predictor for the presence of dysplasia (OR 11.14), CD34 expression (OR 3.60), and PCNA (OR 3.29). Interobserver agreement for this classification was substantial (κ = 0.66).

A simplified CP classification based on observation with ME-NBI is presented. Our results indicate that the classification may be useful for surveillance of BE with high malignant potential.

## INTRODUCTION

The incidence of esophageal adenocarcinoma arising from Barrett esophagus (BE) has been rapidly increasing in many Western countries over the past few decades.^1–3^ It also leads to increasing the rate of hospitalization for esophageal adenocarcinoma and causes a serious problem.^4^ Endoscopic surveillance of BE, the currently accepted standard, aims to reduce morbidity and mortality by early detection and endoscopic therapy of dysplasia or cancer.^5–8^ Current guidelines from gastroenterology societies recommend endoscopic surveillance of BE using white light endoscopy (WLE) with targeted biopsies of any endoscopically visible lesions and 4 random quadrant biopsies from every 2 cm of the BE segment (Seattle protocol).^7,9^ However, it has been pointed out that this protocol has several limitations, such as time required, low compliance, and increased risk of sampling error.^10^ Therefore, establishment of a more effective surveillance program for detecting dysplastic lesions or those with high malignant potential in BE patients is highly desirable.

Recent technological advances in new endoscopic imaging techniques, including chromoendoscopy, magnification endoscopy, autofluorescence imaging, and narrow band imaging (NBI), have provided tools for better identification of specialized intestinal metaplasia (SIM), dysplasia, and early cancer in patients with BE,^11^ with magnifying endoscopy with NBI (ME-NBI) shown to be one of the most promising for accurate endoscopic diagnosis corresponding to histology findings.^12–14^ This modality enables detailed inspection of mucosal morphology without the use of staining agents, and is now used worldwide for diagnosis and surveillance in patients with BE, not only by expert endoscopists but also by nonexperts.^15^ It is considered that surveillance protocols for patients with BE will soon drastically change by employing this new technique.^16^

Several classification systems have been developed for evaluation of BE using ME-NBI on the basis of the detailed characterization of both mucosal and microvascular patterns, and aim to predict the underlying histology to detect early neoplastic lesions.^12–14,17^ Although the usefulness of these classifications has been reported with good accuracy and interobserver agreement, that has not been shown in subsequent validation studies mainly due to their complexity.^18^ Therefore, none is widely used in clinical settings and a standard protocol remains to be established.

Most systems used to classify ME-NBI findings in BE consist of multiple subclassifications, including 4 or more combinations of mucosal patterns and capillary patterns (CPs).^12–14^ Notably, classification of mucosal patterns is too complicated to be used in daily clinical practice. To overcome these shortcomings and establish a simplified classification using ME-NBI findings for stratification of malignant potential in BE, we focused on CP and not mucosal pattern. On the basis of our results, we propose a new CP classification assessed by ME-NBI, which is composed of only 2 categories based on shape and microvessel occupied areas. The aim of the present study was to establish a simplified classification of mucosal morphology focusing only on CP for detecting SIM and dysplasia, as well as markers related to malignant potential in BE patients. Furthermore, we investigated diagnostic concordance when this classification was applied in routine clinical practice.

## MATERIALS AND METHODS

### Patients

We enrolled 108 consecutive BE patients (lesions ≥ 0.5 cm) older than 18 years old who underwent endoscopic examinations from July 2011 to December 2012. Patients who did not have an indication for biopsy (uncontrolled coagulopathy, anticoagulant therapy, esophageal varices, and other serious conditions) were excluded. The protocol of this study was approved by the ethics committee of the Shimane University School of Medicine. A written informed consent was obtained from all the patients.

### Endoscopy

Sedation was performed with intravenous midazolam and all endoscopic examinations were performed by a single-expert endoscopist (GU) using an ME-NBI endoscope (GIF-H260Z, Olympus Medical Systems Co, Tokyo, Japan). A disposable transparent hood (MB-46, Olympus Medical Systems Co) was attached approximately 2 mm distal from the tip of the endoscope for the purpose of maintaining the focal distance during the procedure. After insertion of the endoscope, recognized BE was cleaned with water containing a small amount of 0.04% dimethicone solution (Gascon, Kissei Pharmaceutical Products Inc, Tokyo) to rid the surface of adherent mucus. The esophagogastric junction was defined as the proximal margin of the gastric folds,^19^ and the extent of BE was defined according to the Prague C and M criteria.^20^ BE with greater than 3 cm of circumferential length (*C* ≥ 3.0) was defined as long segment BE (LSBE), whereas others were defined as short segment BE (SSBE). The examined BE areas were randomly selected at the direction of the endoscopist and observed in fully zoomed images to evaluate CP. Next, the areas were cleaned again by 50 mL of water and pronase (20,000 units). Then, enough amount of 0.03% crystal violet (CV) was applied to the BE areas with a spray tube; 3 minutes after spraying the dyes, the sprayed areas were washed thoroughly with water. The pit pattern of the same area was observed without magnification.^21^ Finally, biopsy specimens were obtained from the observed lesions using standard biopsy forceps (Radial Jaw III, Boston Scientiﬁc Co, Natick, MA). Reflux esophagitis grade,^22^ presence of hiatal hernia,^23^ and gastric mucosal atrophy^24^ were determined in each case during the endoscopic examination.

### Classification of Capillary and Pit Pattern

**Figure 1:**
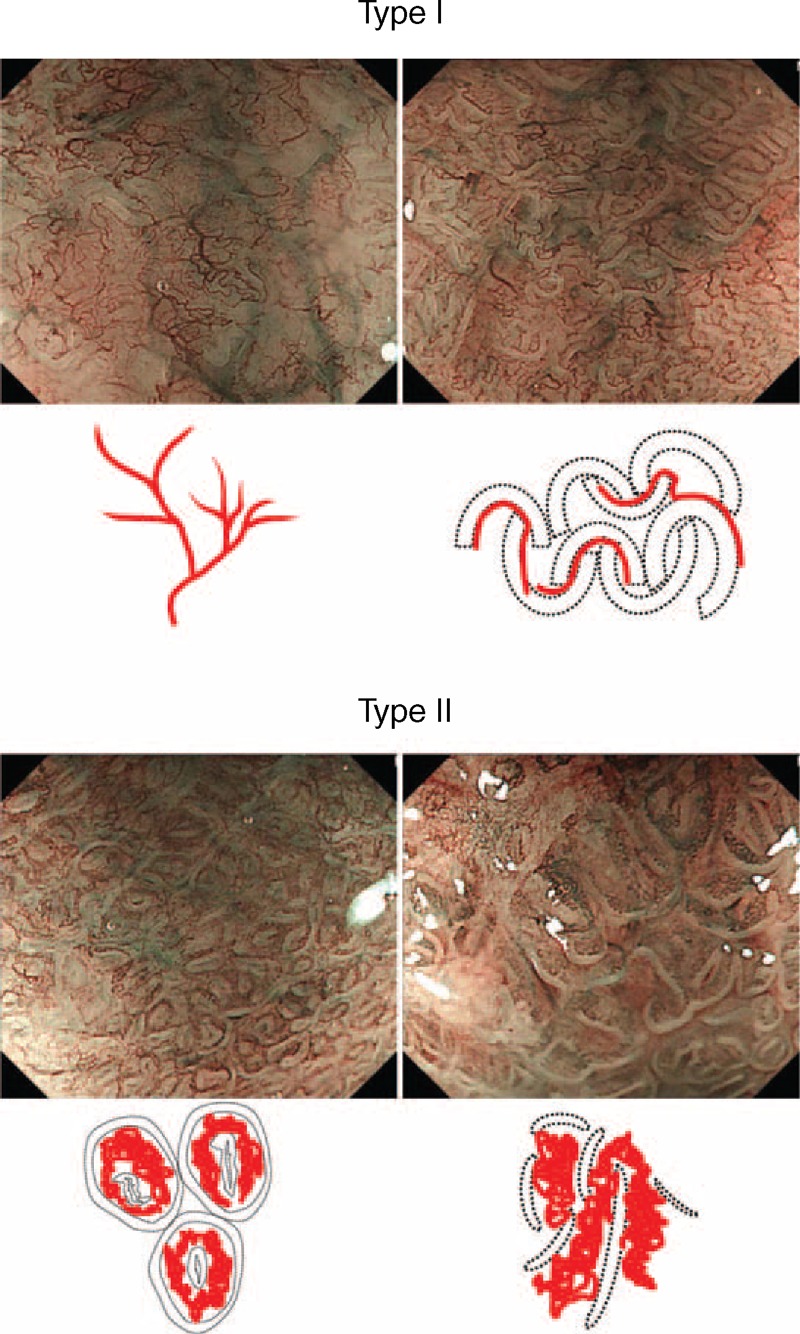
Representative ME-NBI images and schema of simplified classification of capillary pattern in Barrett esophagus. Capillary pattern was divided into type I, a branched or vine-like pattern with a clear shape that is able to be smoothly traced, and type II, a coiled or spiral pattern with a disorderly shape that is not able to be sufficiently traced.

**Figure 2:**
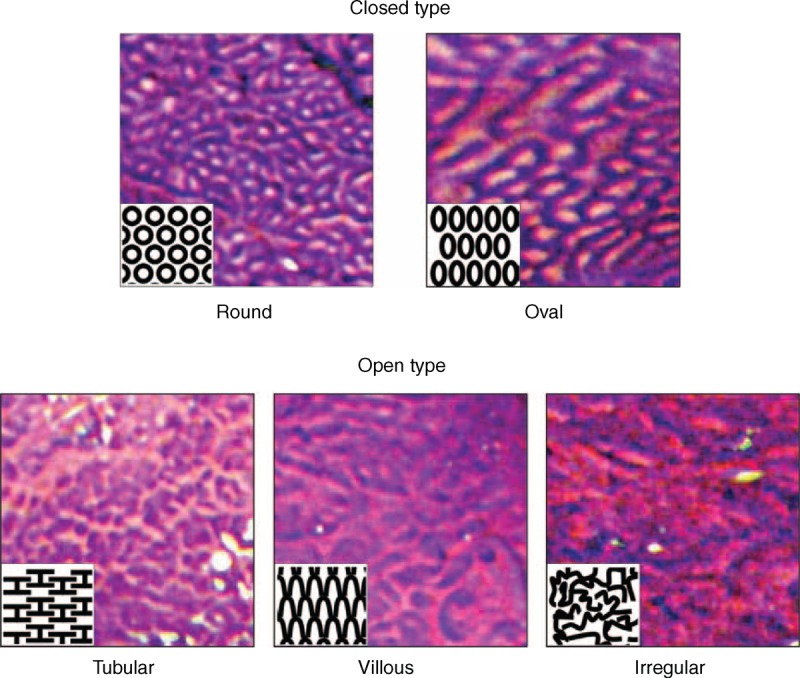
Representative endoscopic images with schema of pit pattern classification by endoscopy with crystal violet staining. Pit pattern was divided into closed type, consisting of round and oval pits, and open type, consisting of tubular, villous, and irregular pits, as previously described.^21^

CP classification was determined on the basis of the shape of the microvessels and their occupied area. To simplify the classification, CP was divided into the following two categories: type I, uniform branched or vine-like pattern with a clear shape that is able to be traced smoothly, and type II, coiled or spiral pattern with a nonuniform shape that cannot be traced sufficiently and with increased vascularity (Figure 1). CP was determined independent of mucosal pattern. Irregular CP frequently observed in mucosa with esophageal adenocarcinomas was included in type II. In addition, pit patterns shown by CV chromoendoscopy were classified into closed and open types, according to the surface network as previously reported.^21,25,26^ The closed type consisted of circular pit patterns, such as round and oval, whereas the open type was used to indicate all other patterns, such as tubular, villous, and irregular (Figure 2). These patterns were judged by 3 expert endoscopists (GU, NI, TY), each of whom had ample experience with ME-NBI examinations of BE patients.

### Histopathology

All biopsy specimens were routinely processed, and stained with hematoxylin and eosin according to standard procedures, and then reviewed by expert gastrointestinal pathologists blinded to the endoscopic findings. Each review was performed according to the revised Vienna classification,^27^ and dysplasia was included as both high and low grades in this study.

**Figure 3:**
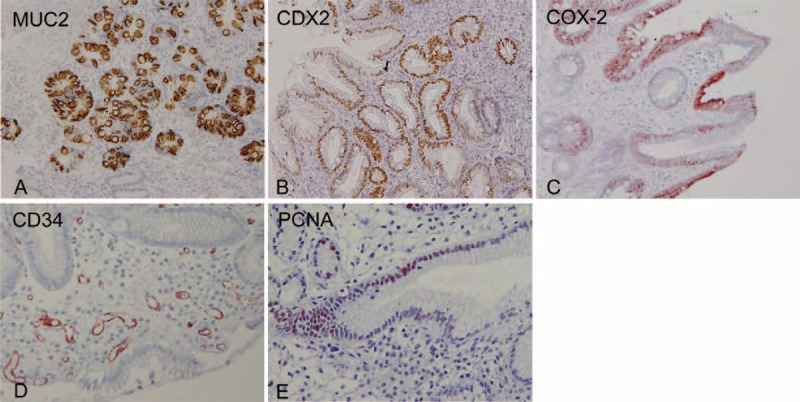
Representative images showing positive expressions of factors related to carcinogenesis. (A) MUC2, (B) CDX2, (C) COX-2, (D) CD34, (E) PCNA. Original magnification x 200.

For each biopsy specimen, the mucin phenotype of Barrett mucosa was determined. Antibodies against human gastric mucin (45MI, 1:100, Novocastra, Newcastle, UK) and MUC2 (Ccp58, 1:100, Novocastra) were used for phenotyping, as previously described.^28–30^ When Barrett epithelial cells were more dominantly stained by the anti-MUC2 antibody than the antihuman gastric mucin antibody, the case was diagnosed as BE with the intestinal predominant mucin phenotype (Figure 3A). Caudal-type homeobox transcription factor 2 (CDX2) (CDX2-88, 1:100, BioGenex, San Ramon, CA) was used as immunostaining markers of intestinal metaplasia, and judged to be positive when the stained area was greater than 50% (Figure 3B).^28,29^

COX-2 protein expression was investigated using an immunohistochemical technique with a specific mouse antihuman COX-2 monoclonal antibody (160112, 1:50, Cayman Chemical, Ann Arbor, MI). COX-2 immunostaining was judged to be positive when the area of staining covered more than 25% of the epithelial cells (Figure 3C).^30,31^ Microvessels were stained with mouse monoclonal antihuman CD34 antibody (N7165, Dako Cytomation, Kyoto, Japan) (Figure 3D), and the grade of angiogenesis in Barrett mucosa was judged according to a method reported by Weidner et al.^32^ Briefly, the density of annular microvessels was evaluated and scored from 1 to 4. BE with a CD34 score of 3 or 4 was defined as having a high grade of angiogenesis.^33^ Cellular proliferation in Barrett epithelium was evaluated by counting the number of cells stained by antiproliferating cell nuclear antigen (PCNA) antibody (PC 10, 1:75, Dako Cytomation). PCNA-stained cells in at least 10 glands were counted and the PCNA index was expressed as the average number of stained cells per gland with Barrett mucosa (Figure 3E).^28,30^

### Percentage of Microvascular Density

**Figure 4:**
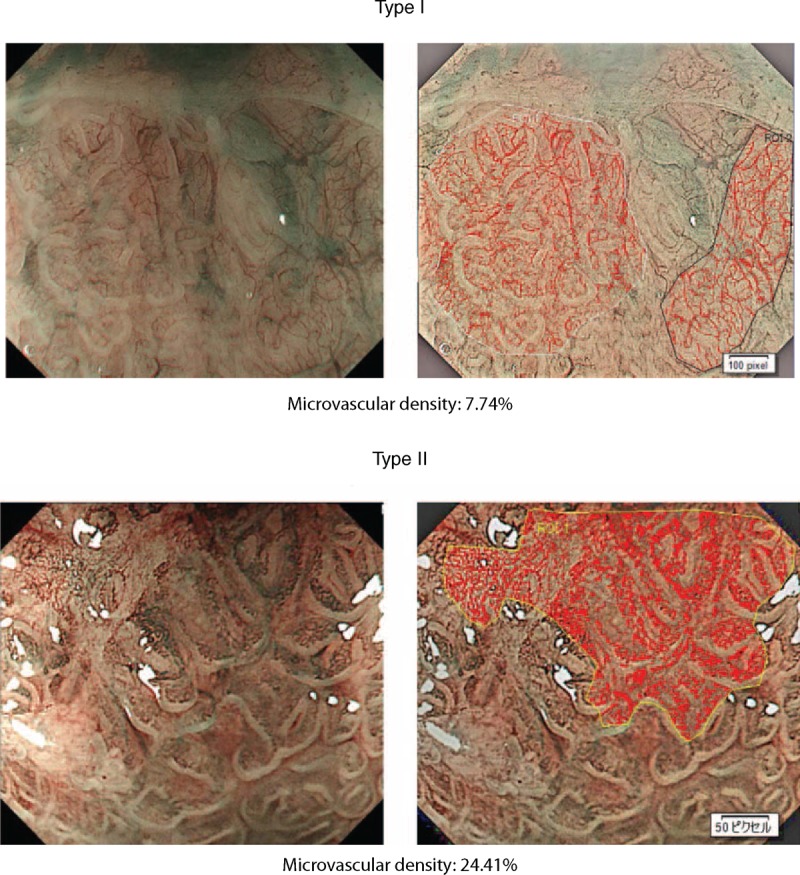
Representative images of microvascular density for each category of capillary pattern. Microvascular density was calculated by the percent area of microvessels in the lesion of interest by ME-NBI using image analysis software. The red line shows vessels selected from the image processed area based on a single-threshold level (hue, saturation, and luminance).

Microvascular density, defined as the percentage area occupied by a vascular bed within the whole area of focus observed by ME-NBI, was calculated using image analysis software (cell Sense, Vers. 1.5^®^, Olympus Medical Systems Co) as follows. First, the in-focus area was segmented from the full zoomed image of the lesion from which the biopsy sample was taken. Next, shading compensation to equalize the brightness of the image and differential contrast enhancement to enhance the contrast of the image were done for easy detection of microvessels in the segmented area. Then, the vessels were selected from the image-processed area based on a single-threshold level (hue, saturation, and luminance). Finally, the percentage of vessel area in the segmented area was calculated (Figure 4).

### Assessment of Interobserver Variability

Interobserver agreement for CP classification was determined using κ statistics. Images taken by ME-NBI were evaluated by 4 endoscopists with extensive experience with ME-NBI (defined as experts) and 4 endoscopists with no such experience (nonexperts), each of whom had previously received board certification from the Japan Gastroenterological Endoscopy Society. All raters were blinded to all other endoscopic and histopathological findings. The present novel classification was explained to them using 20 images from each typical case. Thereafter, 65 randomized images were evaluated and classified into type I and type II by each rater, who was allowed to view the image as many times as necessary before making the classification decision. Assessments by a large number of observers with multiple objectives were analyzed using the method proposed by Siegel and Castellan.^34^ Kappa coefficients of reliability were determined plus 95% confidence intervals (CIs). Interpretation of the κ-value was generally accepted, with a κ-value below 0.20 considered to be poor, 0.21–0.40 fair, 0.41–0.60 moderate, 0.61–0.80 substantial, and 0.81–1.00 very good.

### Statistical Analysis

Chi-square and Mann–Whitney *U* tests were used to examine significant differences among the data. After identification of significant predictors by univariate analysis, the multivariate logistic regression analysis was performed to evaluate independent predictors for dysplasia, angiogenesis (high-grade expression of CD34), and cellular proliferation (PCNA index). Because the PCNA index is a continuous variable, it was transferred to a categorical variable by distinguishing high PCNA index, which was higher than or equal to the median of PCNA, from low PCNA index, which was lower than the median. *P*-values less than 0.05 were considered to indicate signiﬁcance. All statistical analyses were performed using statistical analysis software (SPSS, version 22.0 for the PC, Chicago, IL).

## RESULTS

### Patient Characteristics

**Figure 5:**
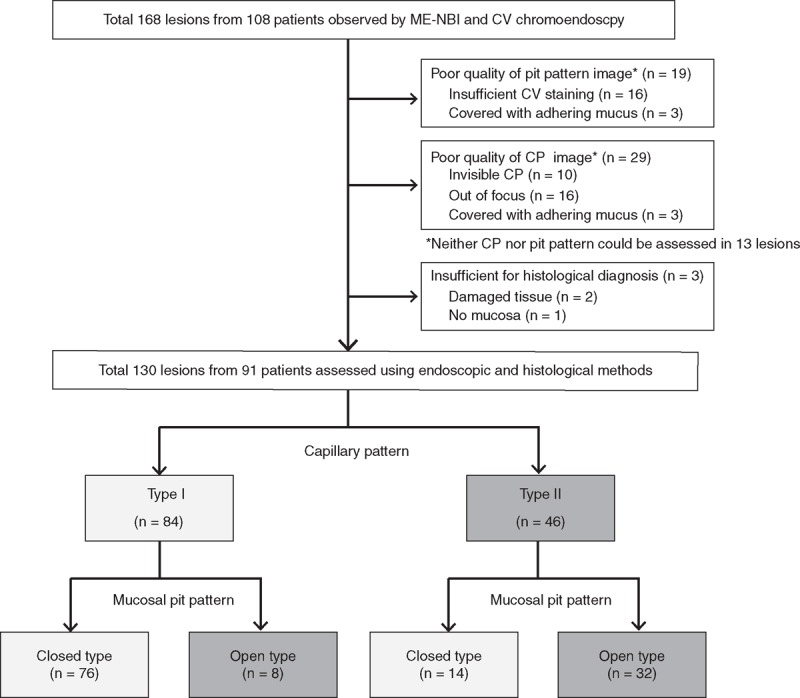
Flow diagram of patients enrolled in the study. One hundred sixty eight lesions from 108 patients were enrolled, of which 38 lesions were excluded. Finally, 130 lesions from 91 patients were evaluated for capillary and pit patterns, as well as histological findings.

In the 108 patients with BE, 168 images of CP were obtained by ME-NBI, of which 130 from 91 patients were considered to be of sufficient quality and eligible for assessment in the present study. The 38 areas excluded from evaluation were disqualified because of either suboptimal image quality or suboptimal biopsy specimens (Figure 5). Patient characteristics, including age, sex, length of BE, presence of reflux esophagitis, gastric mucosal atrophy, and hiatal hernia, were not different between cases with type I and type II CP (Table 1). Of the 130 analyzed lesions, 84 were shown to be type I and 46 to be type II by CP classification, whereas 90 were classified as closed-type pit pattern and 40 as open type by CV chromoendoscopy findings.

**Table 1:**
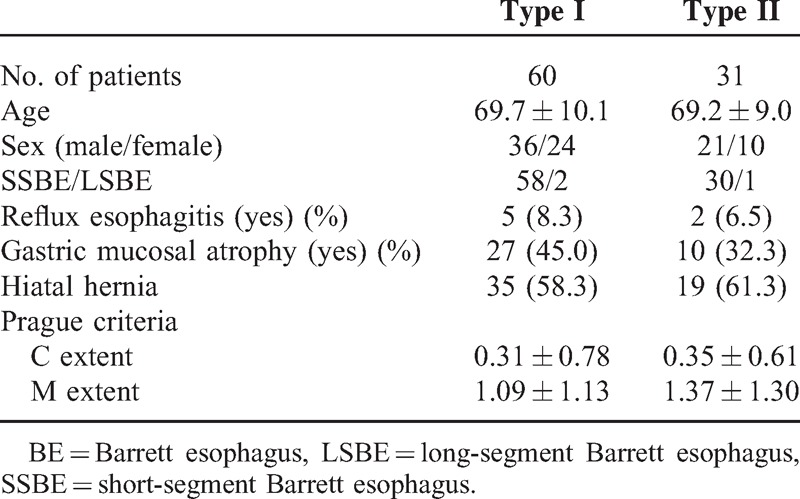
Patient Characteristics

### CP and Mucosal Pit Pattern

We initially investigated the relation between CP and mucosal pit pattern. In a previous study, we proposed a pit pattern classification in BE using CV chromoendoscopy consisting of two types (closed and open), with the open type considered to be closely correlated to SIM and dysplastic Barrett lesions.^21,25,26^ Eight (9.5%) of the type I and 32 (69.6%) of the type II cases were open type, indicating that type II CP was more closely associated with the open-type pit pattern, which occurs more frequently with dysplastic Barrett lesions (*P* < 0.001) (Table 2).

**Table 2:**
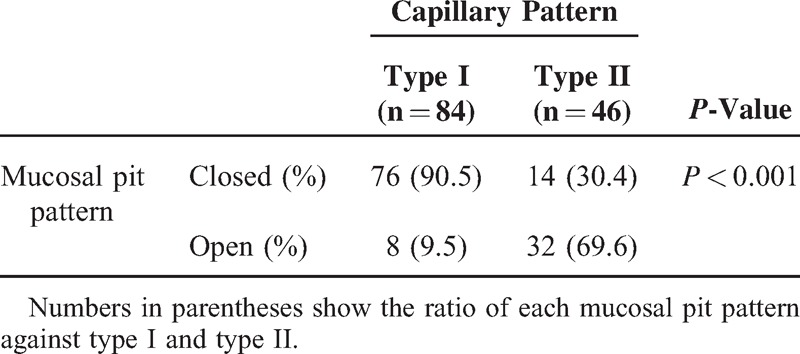
Relationship Between Capillary Pattern and Mucosal Pit Pattern

### CP and Factors Predisposing to Esophageal Adenocarcinoma

Next, we assessed the relation between CP and factors that appeared to contribute to development of esophageal adenocarcinoma. The presence of dysplasia and SIM is highly suggestive markers indicating predisposal to esophageal adenocarcinoma.^35–37^ In addition, it is generally accepted that inflammation, upregulated angiogenesis, and cellular proliferation play important roles in the process of the metaplasia–dysplasia–carcinoma sequence in BE.^38,39^ COX-2 expression, one of the most important inflammation-related factors, could lead to cellular proliferation, angiogenesis, and resistance to apoptosis through the intermediary of prostaglandin E_2_.^40,41^ Thus, we evaluated CP pattern and factors related to malignant potential, including the presence of dysplasia, intestinal metaplasia (assessed by the presence of SIM, intestinal mucin phenotype, and CDX2 expression), and expressions of COX-2 and CD34 as a marker of angiogenesis, and PCNA as a marker of cellular proliferation using immunohistochemistry.

All areas with dysplasia (n = 6) had type II CP (*P* = 0.002), whereas 36 (42.9%) with type I and 28 (60.9%) with type II lesions had SIM (*P* = 0.049). Although there was no significant difference regarding intestinal predominant mucin phenotype between types I and II, the prevalence of CDX2-positive cases in type II was significantly higher than that in type I (*P* = 0.022). CDX2 is a highly sensitive marker of intestinal metaplasia; thus, these data indicated that type II CP is significantly associated with dysplasia and intestinal metaplasia in BE as compared with type I (Table 3). In contrast, the prevalence of dysplasia and SIM in the open type only showed a tendency to be higher than that in the closed type (Table 4).

**Table 3:**
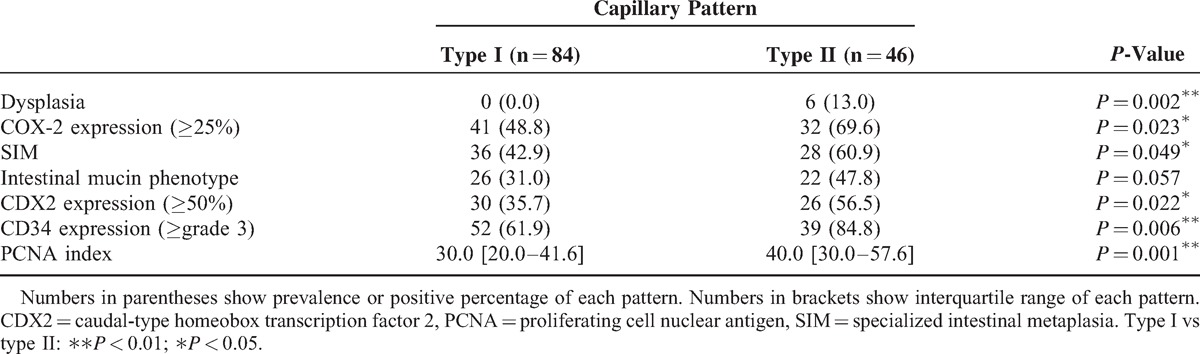
Histological and Immunohistochemical Results (type I vs type II)

**Table 4:**
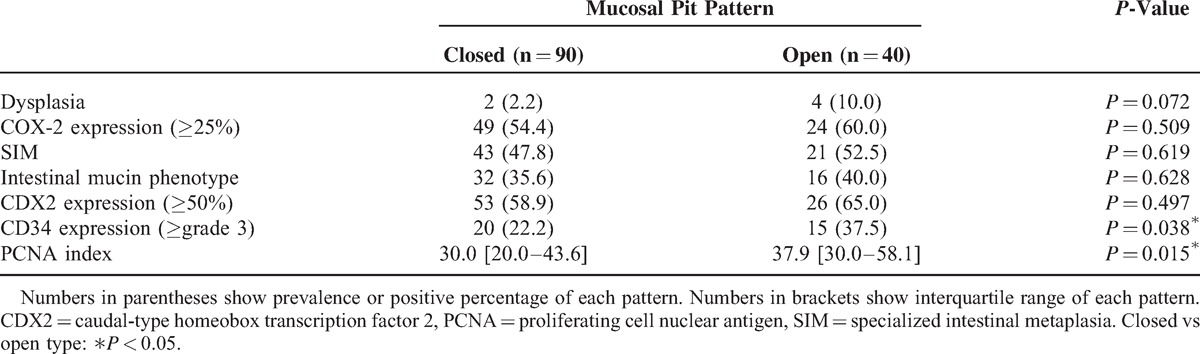
Histological and Immunohistochemical Results (Closed vs Open Type)

COX-2 was positive in 41 lesions (48.8%) in type I and 32 lesions (69.6%) in type II (*P* = 0.023). As for CD34, 52 lesions (61.9%) in type I and 39 lesions (84.8%) in type II showed a high grade of angiogenesis (*P* = 0.006). Furthermore, the median PCNA index value for lesions from type II CP was 40.0 [interquartile range (IQR) 30.0–57.6], which was significantly higher than the value for those from type I (*P* < 0.001) (Table 3). There was also a significant difference between the closed and open types for positive rate of high-grade expression of CD34 (*P* = 0.038) and PCNA index (*P* = 0.015), whereas a trend similar to that seen for CP was recognized with the other factors (Table 4).

### CP and Microvascular Density

**Figure 6:**
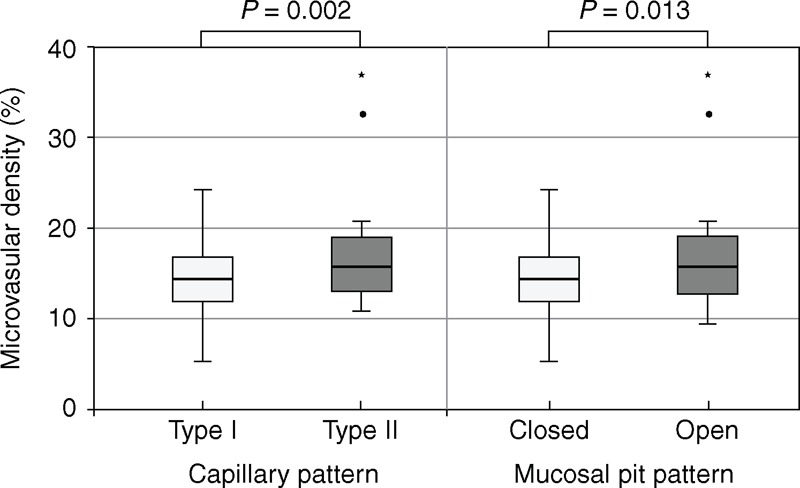
Determination of percentage of microvascular density in lesion of interest. Type II and open type pit pattern showed significantly greater microvascular density than type I and closed type (*P* = 0.002, *P* = 0.013, respectively).

The classification of CP was based on shape and occupied microvessel area. To objectively examine the difference in microvascular density between type I and type II CP, we used image analysis software. The median percentage values for microvascular density were 13.9% (IQR 11.4–16.4) for type I, 16.0% (IQR 12.7–19.0) for type II, 14.1% (IQR 11.5–16.4) for closed type, and 15.4% (IQR 12.8–19.6) for open type. Type II and open type had significantly greater microvascular density than type I (*P* = 0.002) and closed type (*P* = 0.013), respectively (Figure 6).

### Multivariate Analysis for Prediction of Factors Related to Carcinogenesis

To find risk factors for the presence of dysplasia, cellular proliferation (PCNA), and angiogenesis (CD34), multivariate logistic regression analysis was performed. We selected factors that could be evaluated with endoscopy without a biopsy sample, including age, sex, presence of reflux esophagitis, gastric mucosal atrophy, hiatal hernia, length of BE (C-extent, M-extent by Prague criteria), CP (type II), pit pattern (open type), and microvascular density. Multivariate analysis revealed that the presence of type II CP [odds ratio (OR) 11.14; 95% CI 1.20–103.49; *P* = 0.034] and male sex (OR 1.65; 95% CI 1.00–2.74; *P* = 0.050) were independent predictors of dysplasia, the presence of type II CP (OR 3.60; 95% CI 1.38–9.35; *P* = 0.009) and age (OR 0.93; 95% CI 0.87–0.97; *P* = 0.001) were independent predictors of high-grade expression of CD34, and the presence of type II CP (OR 3.29; 95% CI 1.51–7.19; *P* = 0.003) and male sex (OR 1.30; 95% CI 1.03–1.63; *P* = 0.028) were independent predictors of higher PCNA index (Table 5).

**Table 5:**
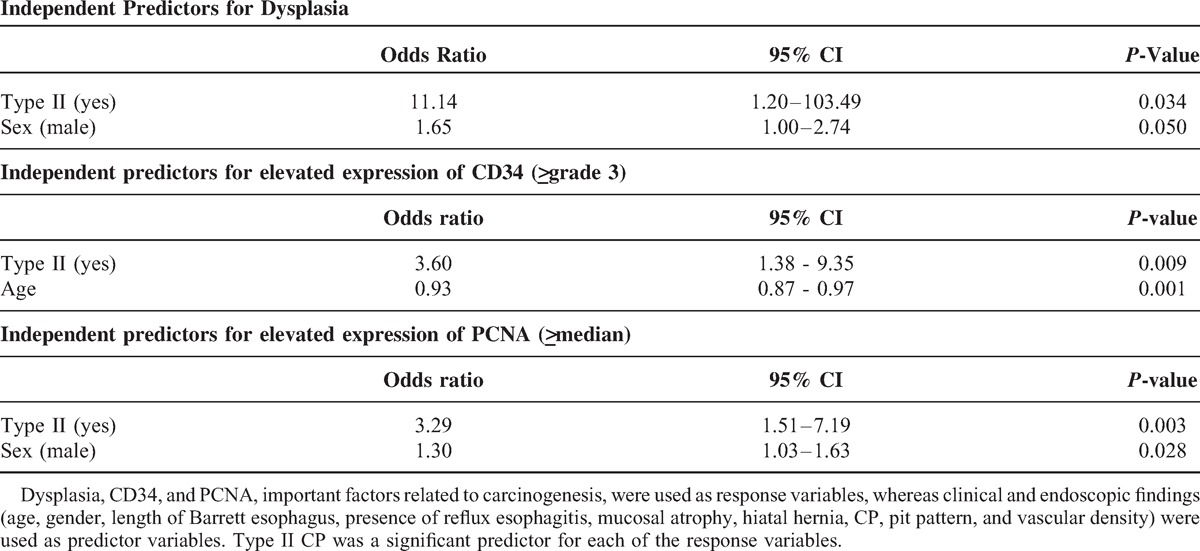
Results of Multivariate Analysis

### Interobserver Agreement Regarding Capillary Pattern

**Figure 7:**
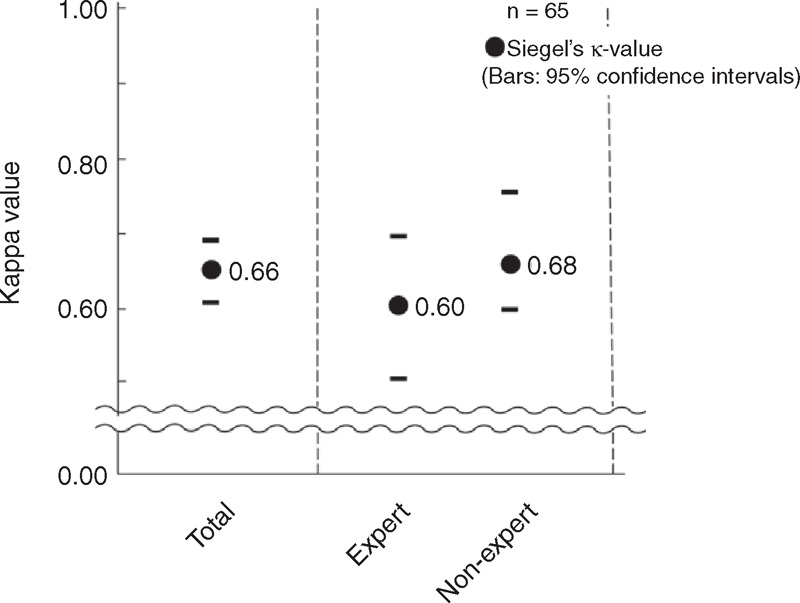
Interobserver agreement for capillary pattern. The κ-value of CP classification was substantial [κ = 0.66 (95% CI 0.62–0.70)] for all raters. The κ-values for the expert and nonexperts were fair to substantial [κ = 0.60 (95% CI 0.50–0.70) and 0.68 (95% CI 0.60–0.75), respectively]. There were no significant differences between these groups.

Finally, we evaluated interobserver agreement regarding our novel classification. The κ-value of the simplified classification using ME-NBI was substantial [κ = 0.66 (95% CI 0.62–0.70)] for all raters. For each of the experts and nonexperts, the κ-value was fair to substantial [κ = 0.60 (95% CI 0.50–0.70) and 0.68 (95% CI 0.60–0.75), respectively] (Figure 7). There were no significant differences between the groups, suggesting that this classification is readily available for clinical practice, irrespective of endoscopist expertise.

## DISCUSSIONS

This is the first study to evaluate the diagnostic value of CP classification in BE by comparing with mucosal pit pattern. Mucosal pit pattern diagnosis using chromoendoscopy has been frequently reported to improve detection of dysplastic lesions in BE as compared with WLE, including our previous studies. ^21,26,42–44^ However, assessment of BE by chromoendoscopy is limited by its labor-intensive and operator-dependent procedures, and use of staining agents that do not always distribute equally across the area of interest. In addition, 23.1% of reported cases were missing a mucosal pit pattern or could not be evaluated.^14^ Therefore, ME-NBI is now used for endoscopic evaluation of BE, and a recent meta-analysis by Mannath et al showed that ME-NBI has high diagnostic precision in detecting high-grade dysplasia with a sensitivity and specificity of 96% and 94%, respectively.^17^ Although the favorable report regarding ME-NBI based classification systems for BE has been published, none of these classifications has been in widespread use mainly because of their complexity.^18,45^ In the present study, we developed the most simplified classification focusing only on CP not mucosal pattern. The strength of our classification is discussed in greater detail below.

In this study, we first compared between CP and mucosal pit pattern for diagnosis. Our results suggested that classification with each modality is useful for BE surveillance. Furthermore, we confirmed that the prevalence of dysplasia in BE tended to be higher in areas with an open type as compared with closed-type pit pattern. However, not all areas with dysplasia could be identified by the pit pattern. On the other hand, all areas with dysplasia had type II CP, indicating that CP classification is more sensitive to detect dysplastic areas in BE. Thus, we concluded that CP assessed by ME-NBI is a more accurate predictor of the presence of dysplasia in BE than mucosal pattern assessed by CV chromoendoscopy or ME-NBI.

Angiogenesis has been linked to formation of CP and carcinogenesis from esophageal adenocarcinoma.^33,46,47^ In addition, Konda et al. demonstrated the process of histological stepwise increases in microvascular density from nondysplastic BE to low-grade dysplasia, then high-grade dysplasia, and finally to cancer.^48^ Their report strongly supports the hypothesis that angiogenesis has a key role in metaplasia–carcinoma progression. In this study, microvascular density and grade of CD34 expression were greater in areas with type II CP than those with type I. This observation suggests that subjectively diagnosed areas with type II CP have high microvascular density that can be determined in an objective manner. Taken together, we consider that using CP classification linked with microvascular density is an appropriate and valid method for detecting dysplastic lesions in BE.

An important strength of our study is that markers related to malignant potential, such as COX-2, CD34, and PCNA, were evaluated by immunohistochemistry for each category of the classification. The relation between CP by ME-NBI and these markers has never been evaluated in the previous studies.^12–14,17^ Elevated expression rates of these markers suggest the presence of inflammation, angiogenesis, and high cellular proliferation. Multivariate analysis showed that the presence of type II CP was the only statistically significant predictor for the presence of dysplasia, high CD34 expression, and PCNA index. Therefore, type II CP might indicate not only dysplasia, but also mucosa with a high malignant potential with increased COX-2, CD34, and PCNA levels. In other words, areas with type I CP may be recognized as areas with lower malignant potential than type II CP, suggesting that further assessment using a biopsy procedure could be avoided in areas with type I. Repeated biopsies are costly, can result in scarring of esophageal mucosa, and hamper endoscopic therapy, such as endoscopic submucosal dissection; thus, the present CP classification system may improve the quality of screening and surveillance in BE patients.

Good diagnostic concordance between endoscopists is a pivotal factor for high clinical value of an endoscopic classification. We found that interobserver agreement was good for the present CP classification and better than other classifications.^15,49–51^ Moreover, there were no differences in agreement between experts and nonexperts, suggesting a short learning curve for this classification method. A previously reported classification system that utilizes ME-NBI had lower levels of inter- and intraobserver agreement than the present method even after completion of a dedicated learning program.^52^

There are some limitations to this study. First, the number of patients with dysplasia was fewer than in previous studies. As a result, sensitivity and specificity of detecting dysplastic lesions are not sufficiently addressed. Second, all the NBI images in this study were selected by a single-expert endoscopist, which might have resulted in a selection bias regarding the study set. Third, this study was performed at a single-tertiary referral center. Finally, all the endoscopic images were based on still images, which may differ from the real-time endoscopic assessment.

In summary, we present a simplified CP classification on the basis of observation by ME-NBI. We found this system to be an adequate method for determining microvascular density and CD34 expression, which were useful as predictors for the presence of dysplasia, as well as expressions of COX-2 and PCNA. The strategy of using this simplified system may overcome the shortcomings of previous ME-NBI classifications and lead to early diagnosis of dysplasia with high diagnostic concordance.
